# Molecular mechanisms of template-independent RNA polymerization by tRNA nucleotidyltransferases

**DOI:** 10.3389/fgene.2014.00036

**Published:** 2014-02-17

**Authors:** Kozo Tomita, Seisuke Yamashita

**Affiliations:** RNA Processing Research Group, Biomedical Research Institute, National Institute of Advanced Industrial Science and TechnologyTsukuba, Japan

**Keywords:** tRNA, CCA, template-independent, nucleotidyltransferase, class-I and II

## Abstract

The universal 3′-terminal CCA sequence of tRNA is built and/or synthesized by the CCA-adding enzyme, CTP:(ATP) tRNA nucleotidyltransferase. This RNA polymerase has no nucleic acid template, but faithfully synthesizes the defined CCA sequence on the 3′-terminus of tRNA at one time, using CTP and ATP as substrates. The mystery of CCA-addition without a nucleic acid template by unique RNA polymerases has long fascinated researchers in the field of RNA enzymology. In this review, the mechanisms of RNA polymerization by the remarkable CCA-adding enzyme and its related enzymes are presented, based on their structural features.

## INTRODUCTION

Every tRNA has the CCA sequence at its 3′-terminus (CCA-3′ at positions 74–76; C_74_C_75_A_76_-3′). The CCA-3′ moiety is required for amino acid attachment (aminoacylation) onto the 3′-end of the tRNA by aminoacyl-tRNA synthetases (Sprinzl and Cramer, 1979), and for peptide-bond formation on the ribosome. The CCA-3′ physically interacts with the ribosomal RNA during translation on the ribosome (Green and Noller, 1997; Kim and Green, 1999; Nissen et al., 2000). The CCA-3′ is also required for tRNA quality control. The tandem C_74_C_75_A_76_C_77_C_78_A_79_-3′ sequence, added onto the 3′-end of tRNA, acts as a degradation signal for dysfunctional tRNA molecules (Wilusz et al., 2011).

The CCA-3′ is synthesized and/or repaired by the CCA-adding enzyme, CTP:(ATP) tRNA nucleotidyltransferase (NT), using CTP and ATP as substrates (Deutscher, 1990; Weiner, 2004). The CCA-adding activity is conserved in all three primary kingdoms – archaea, eubacteria, and eukarya (Yue et al., 1996), and is essential in organisms in which some tRNA genes do not encode CCA-3′ (Aebi et al., 1990). The CCA-adding enzymes belong to the NT family, encompassing enzymes as diverse as polyA polymerase (PAP), terminal deoxynucleotidyltransferase (TdT), DNA polymerase β (pol β), and kanamycin nucleotidyltransferase (KanNT; Holm and Sander, 1995; Martin and Keller, 1996, 2007; Yue et al., 1996). Among the NT family members, the CCA-adding enzyme is a remarkable, template-independent RNA polymerase. The enzyme can synthesize an ordered nucleotide sequence, CCA, onto the 3′-end of a specific primer, tRNA, without a nucleic acid template. Moreover, the enzyme is sensitive to register. It recognizes three kinds of tRNAs – tRNA lacking C_74_C_75_A_76_, C_75_A_76_, or A_76_ – and reconstructs the CCA-3′ sequence as needed.

**FIGURE 1:**
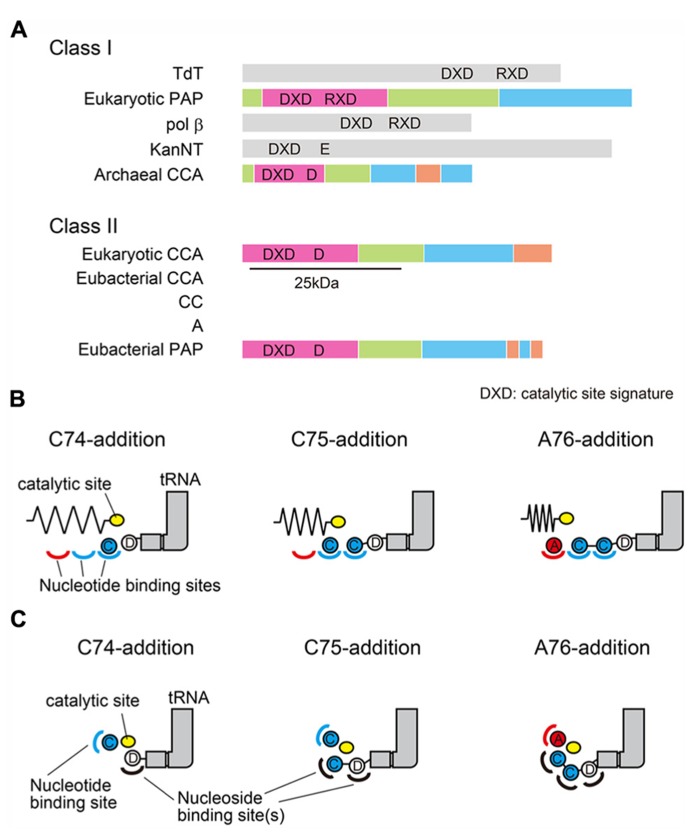
**Two classes of CCA-adding enzymes and the proposed models.** **(A)** Schematic representation of two classes of CCA-adding enzymes and their related enzymes. Archaeal CCA-adding enzymes and eukaryotic polyA polymerases (PAP) belong to class-I. Eubacterial/eukaryotic CCA-adding enzymes and eubacterial PAPs belong to class-II. The N-terminal 25 kDa of the class-II CCA-adding enzyme and PAP are homologous. In some eubacteria, CCA-addition is accomplished by two class-II A-adding and CC-adding enzymes. Domains of CCA (or A, or CC)-adding enzymes and polyA polymerases are colored based on the structures as in **Figure 2A** and **Figure 5A**. DXD is the active site signature containing two carboxylates. The third catalytic carboxylate is also depicted. **(B)** Multiple nucleotide binding sites model. CCA is added by using three nucleotide binding sites (two for CTPs and one for ATP) and a mobile single catalytic site. **(C)** Collaborative templating model. The nucleotide binding site is composed of the refolded 3′-terminus of the RNA and the protein. The nucleotide specificity is collaboratively dictated by the RNA-protein complex.

The CCA-adding enzymes are classified into two classes (class-I and class-II), based on the sequence alignments (Yue et al., 1996). The archaeal CCA-adding enzymes belong to class-I, and share sequence similarity with the eukaryotic PAPs. On the other hand, the eubacterial/eukaryotic CCA-adding enzymes belong to class-II, and share sequence similarity with the eubacterial PAPs (**Figure 1A**). Although both enzyme classes catalyze the same reaction in defined fashions, significant amino acid similarities are not readily apparent between the two classes of CCA-adding enzymes. Only local similarities around the active site signatures have been identified.

In most organisms, the CCA-3′ is synthesized by a single enzyme that can add the CCA-3′ at one time. However, in some eubacteria, the CCA-3′ is synthesized by two distinct, but closely related, class-II enzymes. One adds C_74_C_75_ and the other adds A_76_, and the CCA-3′ is synthesized by these two enzymes in a collaborative manner (Tomita and Weiner, 2001, 2002; Bralley et al., 2005; Neuenfeldt et al., 2008).

For more than forty years, the molecular mechanisms of CCA-addition by the CCA-adding enzyme, a remarkable, template-independent RNA polymerase, and its related enzymes have been a mystery, and have fascinated researchers in the field of RNA enzymology since the identification of their activities. The following two main models had been proposed to explain the unique enzymatic activity, using class-I and class-II CCA-adding enzymes (Sprinzl and Cramer, 1979; Deutscher, 1990; Shi et al., 1998a; Hou, 2000; Li et al., 2000; Tomari et al., 2000; Weiner, 2004). In the first model (**Figure 1B**), there are multiple nucleotide binding sites (two or three) for CTP and ATP, and the active site moves relative to these sites and tRNA during polymerization (Deutscher, 1982; ). In the second model (**Figure 1C**), there is a single nucleotide binding site, and the growing 3′-terminus of the tRNA refolds in the active site to specify nucleotide addition. Thus, the nucleotide specificity is collaboratively dictated by the RNA–protein complex (Yue et al., 1996; Shi et al., 1998a). The second model, termed the “collaborative templating” model, was proposed to explain the biochemical studies showing that there is a single active site in the enzyme for both CTP and ATP incorporations, and the tRNA is fixed on the surface of the enzyme, and neither translocates nor rotates relative to the enzyme during CCA-addition (Shi et al., 1998a; Yue et al., 1998).

Over the past fifteen years, the crystal structures of class-I and class-II enzymes and their complexes with nucleotide(s) and/or various tRNAs or mini-helices have been reported (Li et al., 2002; Augustin et al., 2003; Okabe et al., 2003; Xiong et al., 2003; Tomita et al., 2004, 2006; Xiong and Steitz, 2004; Toh et al., 2008, 2009, 2011; Pan et al., 2010; Yamashita et al., 2014). The structural information has solved most of the long-standing mysteries, but not all yet, about the mechanism of the remarkable, template-independent CCA-adding enzyme and its relatives (Schimmel and Yang, 2004; Weiner, 2004; Xiong and Steitz, 2006). Here, we review the current understanding of the mechanism of CCA-addition by the CCA-adding enzyme, and its related enzymes, based on their crystal structures.

## Class-I ARCHAEAL CCA-ADDING ENZYME

In this section, we review the mechanism of CCA-addition by the class-I archaeal CCA-adding enzyme, based on the crystal structures of the enzyme and its complexes with RNAs and nucleotides or nucleotide analogs (Okabe et al., 2003; Xiong et al., 2003; Xiong and Steitz, 2004; Tomita et al., 2006; Toh et al., 2008; Pan et al., 2010).

### THE TEMPLATE DOES NOT RESIDE WITHIN THE class-I CCA-ADDING ENZYME

**FIGURE 2:**
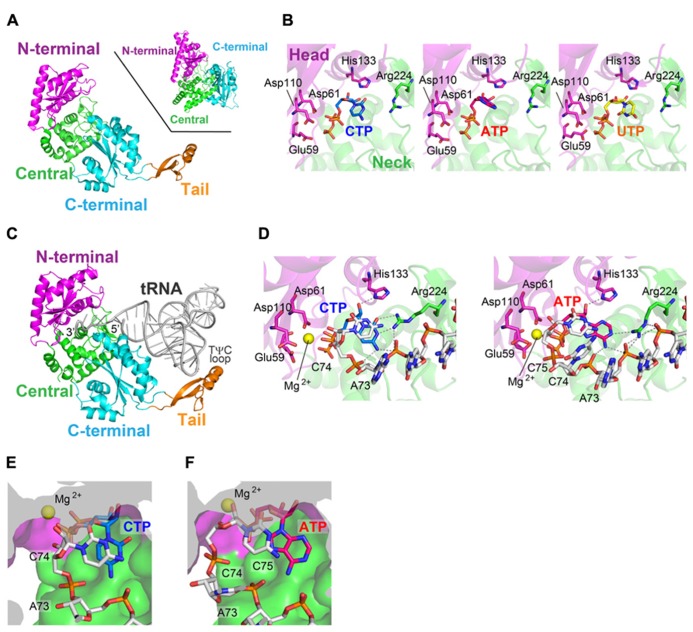
**Structure of the class-I CCA-adding enzyme.** **(A)** Overall structure of *Archaeoglobus fulgidus* CCA-adding enzyme (AFCCA). The N-terminal, central, C-terminal and tail domains are colored magenta, green, cyan, and orange, respectively. For comparison, the structure of eukaryotic polyA polymerase (PAP) is depicted (inset). **(B)** Complex structures of the catalytic core of AFCCA with CTP (left), ATP (middle) and UTP (right). Nucleotides are depicted by stick models. **(C)** Complex structure of AFCCA with tRNA. (**D)** Catalytic core structure at the C_75_-adding stage (left) and the A_76_-adding stage (right). **(E) **Nucleotide binding pocket at the C_75_-adding stage. **(F)** Nucleotide binding pocket at the A_76_-adding stage. The pocket structures in **(E)** and **(F)** are depicted by surface models.

The crystal structures of class-I CCA-adding enzyme from *Archaeoglobus fulgidus* (AFCCA) and its complexes with CTP or ATP were reported (Okabe et al., 2003; Xiong et al., 2003). The structures of AFCCA consist of four domains: N-terminal, central, C-terminal, and tail domains, and the overall structure is U-shaped (**Figure 2A**). This architecture of AFCCA is different from that of the class-II CCA-adding enzyme as described below, and is rather similar to that of the eukaryotic PAP (Bard et al., 2000; Martin et al., 2000). This was anticipated from the comparisons of their primary amino acid sequences (Yue et al., 1996; **Figure 1A**).

The N-terminal domain of AFCCA consists of five β-strands and two α-helices, and three catalytic carboxylates (Glu_59_, Asp_61_, and Asp_110_) reside on the β-sheets. The three catalytic carboxylates are located in close proximity to each other and coordinate the catalytic Mg^2^^+^ ion. The N-terminal catalytic domain structure of AFCCA is homologous to those of other NT family members, including class-II CCA-adding enzyme, pol β and other polynucleotide polymerases (Sakon et al., 1993; Pelletier et al., 1994; Bard et al., 2000; Martin et al., 2000). This suggested that the catalytic cores of the class-I and class-II CCA-adding enzymes share a common ancestor, together with those of other NT family members, and that the class-I and class-II enzymes both catalyze nucleotidyltransfer by the same metal–ion catalytic mechanism (Brautigam and Steitz, 1998). The central domain consists of four stranded β-sheets, and is topologically homologous to the RNA-recognition motif (RRM) of several RNA-binding proteins, such as ribosomal protein S6 (Okabe et al., 2003; Xiong et al., 2003).

The structures of AFCCA complexed with various nucleotides (Xiong et al., 2003) revealed that the nucleotide sits in the inter-domain region, between the N-terminal and central domains of AFCCA (**Figure 2B**). In the complex structures, all nucleotides bound to the catalytic pocket of the enzyme non-specifically, and the base moieties of the nucleotides were disordered in the complex structures. This observation implied that the nucleotide specificity is not dictated by the enzyme alone in the class-I CCA-adding enzyme. Instead, it suggested that the specificity is dictated by the RNA–enzyme complex, as conceptually suggested in the “collaborative templating” model (Shi et al., 1998a; **Figure 1C**). The detailed mechanism of nucleotide selection by the class-I CCA-adding enzyme was later clarified by the determination of several crystal structures of AFCCA complexed with an RNA primer in the presence of an incoming nucleotide, as described below in detail.

### RNA-PROTEIN TEMPLATE FOR CTP AND ATP SELECTION BY class-I CCA-ADDING ENZYME

The complex structures of AFCCA with a tRNA bearing a CCA-3′ terminus, and with various RNA primers mimicking the top-half of a tRNA molecule (tRNA mini-helix or double-stranded RNA) in the presence or absence of nucleotide, were reported (Xiong and Steitz, 2004; Tomita et al., 2006; Toh et al., 2008; Pan et al., 2010). These complex structures of AFCCA with various RNA primers, representing the sequential CCA adding reactions, revealed the detailed mechanism of nucleotide specificity and the dynamic CCA-adding reaction by the class-I CCA-adding enzyme.

AFCCA recognizes the acceptor-TψC helix, the top-half of the tRNA, and does not interact with the tRNA anticodon region at all (Xiong and Steitz, 2004; **Figure 2C**). This is consistent with a previous biochemical study showing that a mini-helix RNA (and even the double-stranded RNA) corresponding to the top-half of the tRNA can be a primer for CCA-addition by the class-I CCA-adding enzyme (Shi et al., 1998b). The tail domain of AFCCA interacts with the elbow region in the TψC loop of the tRNA. The tail domain functions as an anchor for the tRNA, and prevents the tRNA from dislodging from the enzyme surface during CCA-addition. The 3′-terminus of the tRNA enters the active pocket between the N-terminal and central domains of AFCCA (**Figure 2C**).

The ternary structures of AFCCA with a tRNA mini-helix (or double helix RNA) and an incoming nucleotide CTP (or ATP), representing the C_75_-adding (or A_76_-adding) reaction, were reported (Xiong and Steitz, 2004; Tomita et al., 2006). In the ternary complex structures, the geometries of the incoming CTP (or ATP) and the 3′-OH group of the ribose in the 3′-terminal nucleoside of the RNA, relative to the catalytic carboxylates (Glu_59_, Asp_61_, and Asp_110_) and a Mg^2^^+^ metal, suggested that the structures represent the nucleotide insertion stages of RNA polymerization.

In the ternary complex structure of AFCCA with a tRNA mini-helix ending with C_74_ (or double helix RNA) in the presence of CTP, representing C_75_-addition, the cytosine base of the CTP stacks with the cytosine base of C_74_ at the 3′-terminus of the RNA. The 4-NH_2_ group and the N_3_ atom of the CTP hydrogen-bond with the phosphate groups of C_74_ and A_73_ (discriminator nucleoside) of the RNA primer, and with Arg_224_ in the central domain, respectively (**Figure 2D**). In the ternary structure of AFCCA with the tRNA mini-helix (or double helix RNA) ending with C_74_C_75_ in the presence of ATP, representing the A_76_-adding reaction, the adenine base of the ATP stacks with the cytosine base of C_75_ at the 3′-terminus of the RNA. The 6-NH_2_ group and the N_1_ atom of the incoming ATP form hydrogen-bonds with the phosphate groups of C_74_ and A_73_ in the RNA primer and Arg_224_ in the central domain, respectively (**Figure 2D**). Thus, the templates for CTP and ATP selection by AFCCA were found to be the phosphate backbone of the RNA primer and the protein, rather than solely the protein itself, as implicated (Shi et al., 1998a).

After the C_75_-addition, tRNA mini-helix acceptor stem is fixed on the enzyme surface, and the tRNA mini-helix neither translocates nor rotates relative to the enzyme surface. Then, the 3′-terminus of tRNA mini-helix refolds and retracts into the enclosed active pocket (Xiong and Steitz, 2004; Tomita et al., 2006; Toh et al., 2008; Pan et al., 2010). The refolding of the 3′-terminus of the tRNA in the pocket places the ribose 3′-OH group of C_75_ in the RNA proximal to the active site of the enzyme. The structural changes in the active pocket of the enzyme, as well as the refolding of the 3′-end of RNA, after C_75_-adition, ensure that the active pocket is free of any nucleotide, for successive ATP accommodation in the pocket. Thus, a single active pocket can be utilized for both C-addition and A-addition by the class-I CCA adding enzyme.

These sequential structural analyses of AFCCA also revealed that the size and the shape of the nucleotide pocket, composed of the growing 3′-terminus of the RNA and the enzyme, successively change during the CCA-adding reaction (**Figures 2E,F**). At the C_75_-adding stage, the size and the shape of the nucleotide binding pocket are suitable for CTP accommodation, and the larger ATP cannot snugly fit in the pocket. After C_75_-addition, the 3′-terminus of the RNA refolds in the enclosed active site, and the size and the shape of the nucleotide binding pocket become suitable for ATP. Although the smaller CTP could bind in the pocket, it does not snugly fit (Tomita et al., 2006; Pan et al., 2010). Thus, the nucleotide specificity of the class-I CCA adding enzyme shifts from CTP-specific to ATP-specific during the successive CCA-adding reactions.

Together, these extensive crystallographic analyses suggest that the template of the class-I CCA-adding enzyme is neither the protein nor the RNA, but the RNA–protein complex. The RNA primers neither translocate nor rotate relative to the enzyme surface, and a single active pocket is utilized for both C- and A-addition, by successive refolding of the 3′-terminal nucleoside in the enclosed active pocket. The successive refolding of the 3′-terminus of the tRNA during polymerization changes the nucleotide specificity of the class-I CCA-adding enzyme from CTP to ATP. These structural features explained the previous biochemical studies well (Shi et al., 1998a; Yue et al., 1998).

### DYNAMICS OF CCA-ADDITION BY THE class-I CCA-ADDING ENZYME

**FIGURE 3:**
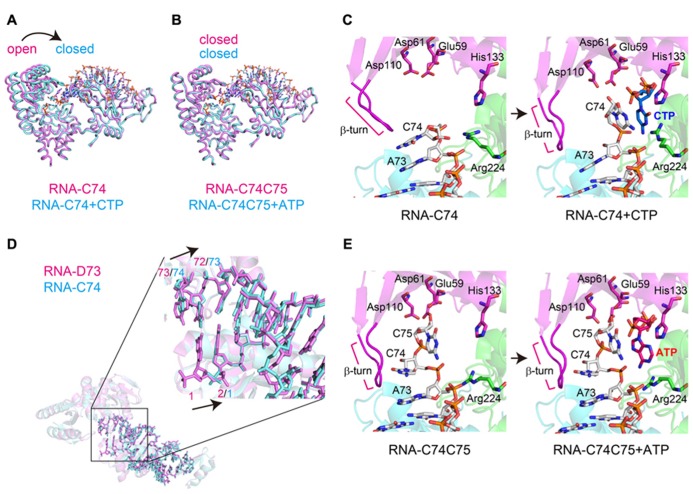
**Dynamics of CCA-addition by the class-I CCA-adding enzyme.** **(A)** Superimposition of the complex structures of AFCCA with a tRNA mini-helix at the C_75_-adding stage, in either the absence (colored magenta) or the presence (colored cyan) of CTP. **(B)** Superimposition of the complex structures of AFCCA with a tRNA mini-helix at the A_7__6_-adding stage, in either the absence (colored magenta) or the presence (colored cyan) of ATP. **(C)** Comparison of the catalytic core structures of AFCCA at the C_75_-adding stages in either the absence (left) or the presence (right) of CTP. For clarity, only the key amino acid residues of AFCCA are shown. RNA and nucleotides are depicted by stick models. **(D)** Superimposition of RNAs at the C_74_-adding stage (colored magenta) and C_75_-adding stage (colored cyan). RNA at the C_74_-adding stage adopts an extended form. **(E)** Comparison of the catalytic core structures of AFCCA at the A_76_-adding stage in either the absence (left) or the presence (right) of ATP. For clarity, only the key amino acid residues of AFCCA are shown. RNA and nucleotides are depicted by stick models.

The binary complex structures of AFCCA with a tRNA mini-helix in the absence of incoming nucleotides were also reported (Tomita et al., 2004; Toh et al., 2008). The comparison of the binary and ternary structures highlights the dynamic change in the orientation of the N-terminal domain of the enzyme and the 3′-terminus of the RNA during CCA-addition (**Figures 3A,B**).

In the binary complex of AFCCA with a tRNA mini-helix ending in either D_73_ (D is the discriminator nucleoside) or C_74_, the nucleobase of the 3′-terminal nucleoside stacks onto the preceding nucleobase, and the ribose 3′-OH group of the 3′-terminal nucleoside is far from the active site (three catalytic carboxylates). This structure represents an inactive form. At the C_7__5_-adding reaction stage, upon binding the incoming CTP in the active site, the N-terminal domain of AFCCA relocates toward the central domain, leading the enzyme to transit from an open conformation to a closed conformation (**Figure 3A**). This allows the 3′-nucleoside C_74_ of the RNA to flip, and positions the ribose 3′-OH group of the 3′-terminal nucleoside proximal to the catalytic residues and the triphosphate group of the incoming CTP. This structure represents a catalytically active form (**Figure 3C**). Although the structure representing C_74_-addition was not determined, the binary complex of AFCCA with tRNA mini-helix ending in D_73_ showed that the 3′-terminal D_73_ of RNA and three catalytic carboxylates well superimposed onto the 3′-terminal C_74_ of RNA and the catalytic carboxylates, respectively, in the binary complex of AFCCA with tRNA mini-helix ending in C_74_, as described below (**Figure 3D**). Thus, C_74_-adding reaction would proceed in the same mechanism as C_75_-adding reaction.

At the C_74_-adding reaction stage, the mini-helix RNA ending with D_73_ adopts an extended form (Tomita et al., 2006; **Figure 3D**). After the C_74_-addition reaction is completed, the N-terminal domain of the enzyme relocates outward, and the enzyme transits to the open, inactive form. Then, the 3′-terminal nucleoside (C_74_) of the tRNA mini-helix flips back into the active pocket, and the acceptor helix of the tRNA mini-helix shrinks back from the extended form. The change in the tRNA mini-helix from the extended form to the shrunken form allows the active pocket to become nucleotide free, for successive CTP accommodation and C_75_-addition. CTP binding in the active pocket for C_75_-addition induces the relocation of the N-terminal domain of the enzyme toward the central domain again (the enzyme transits to an active closed form again; **Figure 3A**). The 3′-nucleoside of the tRNA flips again, and the C_75_-adding reaction proceeds (**Figure 3C**). Thus, in both the C_74_-adding and C_75_-adding reactions, CTP binding in the active site dynamically induces the conformational change of the enzyme from an inactive open form to an active closed form, and only the correct nucleotide, CTP, can allow the transition of the enzyme.

After the C_75_-adding reaction, the enzyme is fixed in an active closed from (**Figure 3B**). The 3′-terminus of the tRNA mini-helix refolds in the enclosed active site, and a newly shaped nucleotide binding pocket is created by the enzyme and the 3′-terminus of the RNA (**Figure 3E**). ATP can bind in the nucleotide pocket, and A_76_-addition proceeds without the open to closed conformational change of the enzyme. The fixation of the enzyme in a closed conformation after C_75_-addition is facilitated by the interaction between the β-turn in the N-terminal domain and the 3′-terminus of the tRNA mini-helix. The mutation of a key amino acid residue, Arg_224_, reduced C_74_C_75_-addition, but not A_76_-addition, *in vitro*, indicating that Arg_224_ in the pocket does not discriminate ATP from CTP in the A_76_-adding reaction (Tomita et al., 2006; Pan et al., 2010). Thus, the A_76_-adding reaction is static, and is distinct from the C_74_C_75_-adding reactions, which are accompanied by the dynamic open to closed conformational transition of the enzyme and require the proper conformation of Arg_244_ in the pocket. Consecutive conformation changes of the β-turn in the N-terminal domain accompany the refolding of the tRNA 3′-terminus during the reaction (**Figures 3C,E**). The β-turn in the N-terminal domain monitors the 3′-terminal sequence of the tRNA for correct CCA-addition (Toh et al., 2008).

After A_76_-addition, the N-terminal domain relocates outward, triggered by pyrophosphate release, and the enzyme adopts the open conformation. At this stage, there is no room to accommodate another nucleotide in the active pocket. Finally, a tRNA with a CCA-3′ terminus dissociates from the enzyme, and the CCA-adding reaction is completed (Xiong and Steitz, 2004; Tomita et al., 2006).

**FIGURE 4:**
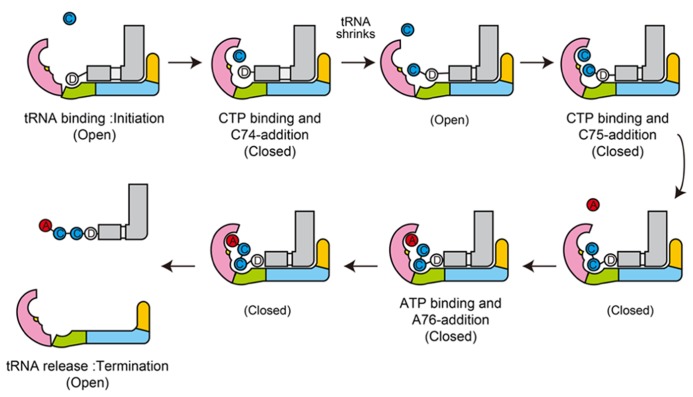
**Mechanism of CCA-addition by class-I CCA-adding enzymes.** Schematic representation of the CCA-adding reaction by class-I CCA-adding enzymes. The N-terminal, central, C-terminal, and tail domains are colored magenta, green, cyan, and orange, respectively. tRNAs are colored gray. Catalytic sites are colored yellow in the N-terminal domains.

The dynamic sequence of the CCA-adding reaction by the class-I CCA-adding enzyme, revealed by the crystallographic analyses of complexes of class-I CCA-adding enzyme with various RNA primers with or without nucleotides, is presented in **Figure 4**.

## THE class-II CCA-ADDING ENZYME AND ITS RELATED ENZYMES

In this section, we review the mechanism of CCA-addition by the class-II eubacterial/eukaryotic CCA-adding enzyme, and its related class-II eubacterial CC-adding and A-adding enzymes, based on the crystal structures of the enzymes and their complexes with RNAs (Li et al., 2002; Augustin et al., 2003; Tomita et al., 2004; Toh et al., 2009; Yamashita et al., 2014).

### PROTEIN-BASED TEMPLATE FOR CTP AND ATP SELECTIONS BY class-II CCA-ADDING ENZYME

The crystal structures of the class-II CCA-adding enzymes from *Bacillus stearothermophilus *(BstCCA), *Thermotoga maritima* (TmCCA) and human mitochondria (HmtCCA) were reported (Li et al., 2002; Augustin et al., 2003; Toh et al., 2009).

**FIGURE 5:**
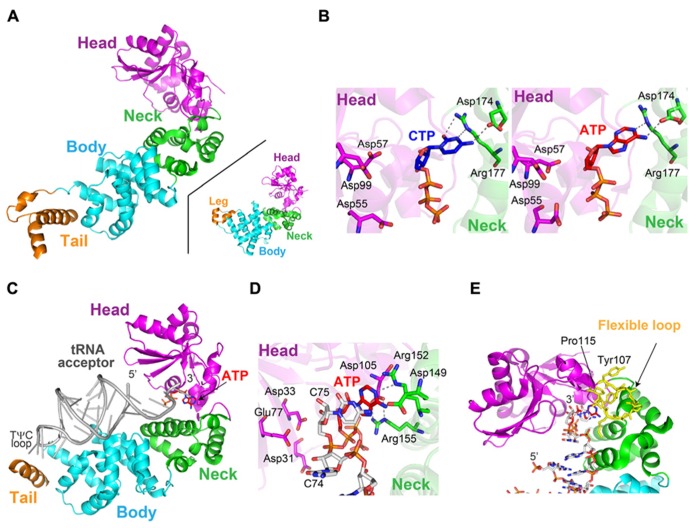
**Structures of the class-II CCA-adding enzyme and its related A-adding enzyme.** **(A)** Overall structure of *Thermotoga maritima* CCA-adding enzyme (TmCCA). The head, neck, body and tail domains are colored magenta, green, cyan, and orange, respectively. For comparison, the structure of *Escherichia coli* polyA polymerase (EcPAP) is depicted (inset). **(B)** Structures of the catalytic pocket of TmCCA with CTP (left) and ATP (right). Nucleotides are depicted by stick models. **(C)** Overall structure of *Aquifex aeolicus* A-adding enzyme (AaL) complexed with tRNA lacking the terminal A_76_ and an ATP analog. The anticodon region in the structure was disordered. **(D)** Structure of the catalytic core of AaL, in the presence of a tRNA primer lacking the terminal A_76_ and an ATP analog. The ATP analog and the tRNA are depicted by stick models. **(E)** The flexible loop (colored yellow, amino acid residues 107–115) in the head domain would interact with the 3′-terminal region of the tRNA. The 3′-terminal region of tRNA and the ATP analog in the AaL complex in **(C)** were modeled onto the structure of TmCCA in **(A)**.

The class-II CCA-adding enzymes adopt a sea-horse-shaped structure, and consist of four domains – the head, neck, body and tail domains (**Figure 5A**). The overall architecture of the class-II CCA adding enzyme is different from that of the class-I CCA-adding enzyme (Okabe et al., 2003; Xiong et al., 2003; **Figure 2A**), and it is rather similar to that of the eubacterial PAP from *Escherichia coli* (EcPAP; Toh et al., 2011; **Figure 5A**). This was anticipated by the comparisons of their amino acid sequences (Yue et al., 1996; **Figure 1**), although EcPAP adopts a sea-otter-shaped structure.

The head domain of the class-II CCA-adding enzyme comprises five-stranded β sheets connected by two α helices, with three conserved catalytic carboxylate residues (Asp_55_, Asp_57_, Asp_99_ of TmCCA) on the β sheets, and the Mg^2^^+^ ions are coordinated by the carboxylates. As described, the head domain structure of the class-II CCA-adding enzyme is homologous to that of the class-I CCA-adding enzyme (**Figure 2A**), together with those of the catalytic domains of other polynucleotide polymerases. The neck domain is composed of helices, and includes the key nucleobase-interacting residues (Asp and Arg) that are putatively conserved among the class-II enzymes. The body and tail domains are composed of a bundle of α helices, and recognize the acceptor and TψC helices of the tRNA, as described below.

In the complex structures of the class-II CCA-adding enzymes (BstCCA and TmCCA) with CTP or ATP, the nucleotide sits in the inter-domain region between the head and neck domains (**Figure 5B**). Both nucleotides are recognized in the same active pocket, through Watson-Crick-like base pairings between the nucleobases and the conserved Asp and Arg residues in the neck domain (Li et al., 2002; Toh et al., 2009). The 4-NH_2_ of CTP and the 6-NH_2_ of ATP form hydrogen-bonds with Asp_174_, whereas the N_3_ atom of CTP and the N_1_ atom ATP hydrogen-bond with Arg_177_. The O_2_ atom of CTP also hydrogen-bonds with Arg_177_ (**Figure 5B**; the amino acid numbering is according to TmCCA).

The mechanism of nucleobase recognition by the class-II CCA-adding enzyme is distinct from that observed in the class-I CCA-adding enzyme (**Figures 2D,E**). The template for the CCA-addition by the class-II CCA-adding enzyme is composed of the protein itself, rather than the RNA–protein complex as in the class-I CCA-adding enzyme. The protein-template of the class-II CCA-adding enzymes was confirmed by the mutations of Asp and Arg in the neck domain. The rational mutagenesis of the two key residues in the neck domain allowed the enzyme to add other nucleotides *in vitro* (Cho et al., 2007).

### PROTEIN-BASED TEMPLATE FOR ATP-SELECTION BY THE class-II A-ADDING ENZYME

Although the structures of the class-II CCA-adding enzyme and its complexes with CTP and ATP are available (Li et al., 2002; Toh et al., 2009), the structures of the class-II CCA-adding enzyme complexed with tRNA (or RNA) have not been reported yet. Thus, the detailed RNA polymerization mechanism of CCA-addition by the class-II CCA-adding enzyme remained enigmatic.

Compounding the unsettled questions on the polymerization mechanism by the class-II CCA-adding enzyme, in some eubacteria such as *Aquifex aeolicus*, the CCA-adding activity is split between two distinct, but closely related, enzymes – one adds C_74_C_75_ and the other adds A_76_ (Tomita and Weiner, 2001, 2002; Bralley et al., 2005; Neuenfeldt et al., 2008; **Figure 1**). In *A. aeolicus*, which is placed at the deepest root of the 16S rRNA-based phylogenetic tree (Pace, 1997), the CC-adding and A-adding enzymes collaboratively synthesize the CCA-3′ (Tomita and Weiner, 2001). On the other hand, in *T. maritima*, which is also located at the deepest root and is evolutionarily close to *A. aeolicus*, a single enzyme homologous to the *A. aeolicus* A-adding enzyme (AaL), rather than the CC-adding enzyme (AaS), adds CCA-3′ (Tomita and Weiner, 2001, 2002).

The complex structure of AaL with tRNA lacking the terminal A_76_ and an ATP analog was reported (Tomita et al., 2004). AaL also adopts a sea-horse-shaped structure, as found with the other class-II CCA-adding enzymes (**Figure 5C**). As anticipated from the sequence similarity and closely related phylogeny (Tomita and Weiner, 2002), the overall structure of AaL superimposed well onto that of TmCCA (Toh et al., 2009).

In the complex structure of AaL with tRNA lacking the terminal A_76_ and an ATP analog (**Figure 5C**), the acceptor and TψC helices of the tRNA are recognized by the enzyme, and the anticodon region does not interact with the enzyme. As in the class-I CCA-adding enzyme (**Figure 2C**), the tail domain of AaL interacts with the elbow region of the tRNA, and functions as an anchor.

The 3′-terminus of the tRNA enters the active pocket, which resides between the head and neck domains of AaL. The geometry of the incoming ATP analog, the 3′-OH group of C_75_ of tRNA, relative to the catalytic carboxylates (Asp_31_, Asp_33_, and Glu_74_), suggested that the structure represents the insertion stage of A_76_addition (**Figure 5D**). The adenine base of the ATP analog is sandwiched by the cytosine base of C_75_ of tRNA and by hydrogen-bonds between Asp_105_ and Arg_155_. Thus, the binding pocket of ATP is composed of the 3′ end of the tRNA and the protein. In the ternary complex structure, as observed in the complex structure of TmCCA (or BstCCA) with ATP (**Figure 5B**), the adenine base is recognized by the side chains of the conserved amino acids, Asp_149_ and Arg_152_, through Watson-Crick-like hydrogen-bonds (**Figure 5D**). The 6-NH_2_ and the N_1_ atom of ATP form hydrogen-bonds with Asp_149_, and Arg_152_, respectively. Thus, the specificity for ATP by AaL is determined by the side chain of the protein itself, as in the class-II CCA-adding enzymes.

Biochemical and genetic studies revealed that a flexible loop in the head domains of the CCA-adding and A-adding enzymes is involved in the A_76_-adding reaction, but not the C_74_C_75_-adding reaction (Tomita et al., 2004; Neuenfeldt et al., 2008; Toh et al., 2009). In most of the reported crystal structures of the class-II CCA-adding and A-adding enzymes, the loop structure is disordered (Li et al., 2002; Augustin et al., 2003; Tomita et al., 2004). The loop region was only clearly visible in the crystal structure of *apo* TmCCA (Toh et al., 2009; **Figure 5E**). The loop extends from the head domain to the neck domain, bridging the two domains. The superimposition of the structures of *apo* TmCCA and AaL complexed with tRNA revealed that the loop would interact with the 3′-part of the tRNA (Toh et al., 2009; **Figure 5E**). It is likely that the loop recognizes the growing 3′-CC sequence of the tRNA, and fixes the conformations of two key residues, Asp_174_ and Arg_177_, for the specific recognition of ATP. The corresponding loops in the CC-adding enzymes are shorter than those in the CCA-adding and A-adding enzymes, and the shorter loop was suggested to be one of the hallmarks of the CC-adding enzyme (Neuenfeldt et al., 2008).

### TRANSLOCATION AND ROTATION OF tRNA DURING CC-ADDITION BY THE CC-ADDING ENZYME

**FIGURE 6:**
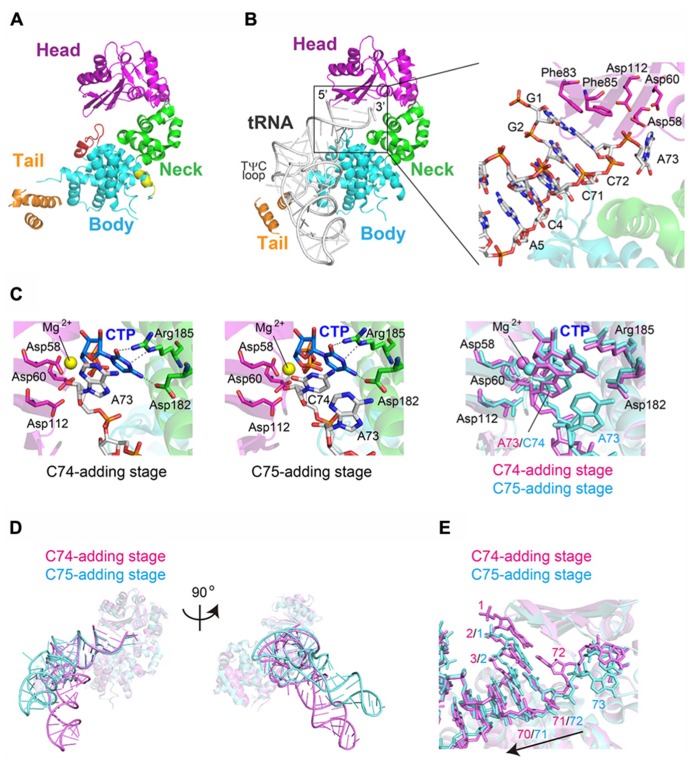
**Structure of the class-II CC-adding enzyme.** **(A)** Overall structure of *Aquifex aeolicus* CC-adding enzyme (AaS). The head, neck, body, and tail domains are colored magenta, green, cyan, and orange, respectively. Unique insertion helices are colored red and yellow. **(B)** Complex structures of AaS with tRNA lacking CCA. The tRNA is depicted by a stick model. Detailed view of the interaction between the acceptor helix of tRNA and AaS (inset). **(C)** Structures of the catalytic pocket of AaS at the C_74_-adding (left) and C_75_-adding (middle) stages. CTPs are depicted by stick models. The superimposition of the structures at C_74_-adding (magenta) and C_75_-adding (cyan) stages (right). **(D)** Comparison of the overall complex structures of AaS at the C_74_-adding (cyan) and C_75_-adding (pink) stages. **(E)** Detailed view of the superimposed structures of the tRNA acceptor helices at the C_74_-adding (cyan) and C_75_-adding (pink) stages in **(D)**.

More recently, the structures of *A. aeolicus *CC-adding enzyme, AaS (Tomita and Weiner, 2001), in its *apo* form and in complexes with various tRNAs were reported (Yamashita et al., 2014). AaS also adopts a sea-horse-shaped structure similar to the other class-II CCA-adding and A-adding enzymes (**Figure 6A**). Although the structures of the head and neck domains of AaS are homologous to those of the class-II CCA-adding and A-adding enzymes, the structure of the body domain of AaS slightly differs from those of the CCA-adding and A-adding enzymes (**Figures 5A** and **6A**). The overall structure of AaS adopts a relatively closed form, by the insertion of an additional α-helix between the head and neck domains, and the body domain of AaS contains an additional α-helix and forms a bulging structure.

The complex structures of AaS with tRNA and an incoming CTP, representing the C_74_-adding and C_75_-adding stages, were also reported (Yamashita et al., 2014; **Figures 6B,C**). As observed in the complex structure of AaL with tRNA (Tomita et al., 2004; **Figure 5C**), AaS also recognizes the top-half region of the tRNA, and does not interact with the anticodon region (**Figure 6B**). The TψC loop and D-loop of the tRNA interact with the tail domain of AaS.

In the C_74_-adding structure, the base-pair at the top of the tRNA acceptor helix stacks with Phe_83_ and Phe_85_ on the β-sheet in the head domain, and the 3′-terminal discriminator nucleoside A_73_ enters the active pocket. The cytosine base of the incoming CTP and the adenine base of A_74_ are stacked, and the triphosphate of the CTP and the ribose 3′-OH group of A_73_ are proximal to the catalytic carboxylates (Asp_58_, Asp_60_ and Asp_112_) and Mg^2^^+^ ion. In the ternary structure, the 4-NH_2_ and the N_3_ atom of CTP form hydrogen-bonds with Asp_182_ and Arg_185_, respectively. The O_2_ atom of CTP also form a hydrogen-bond with Arg_185_ (**Figure 6C**). The mechanism of CTP recognition by AaS is the same as those observed in the complex structures of class-II CCA-adding enzyme with CTP in the absence of tRNA (Li et al., 2002; Toh et al., 2009). In the complex structure of AaS, representing the C_75_-adding stage, the mechanism of CTP recognition is the same as that of CTP recognition at the C_74_-adding stage (**Figure 6C**). Thus, the C_74_ and C_75_-adding reactions both proceed by the same mechanism, using the same active pocket. The size and the shape of the nucleotide binding pocket at the C_74_-adding and C_75_-adding stages are suitable to accommodate CTP, but not the other three nucleotides. CTP is selectively accommodated as a consequence of competition between nucleotides, using the conserved Asp_182_ and Arg_185_ in the pocket.

A comparison of the complex structures of the C_74_-adding and C_75_-adding stages revealed the translocation and rotation of the tRNA relative to the enzyme (**Figure 6D**). As a consequence of its backward translocation, the tRNA rotates and the relative orientation of its anticodon region on the enzyme changes by approximately twenty five degrees, as compared to that of the tRNA in the C_74_-addition stage. In the complex structure, the positions that were formerly occupied by the terminal base-pair (G_1_–C_72_) of the tRNA acceptor stem at the C_74_-addition stage are now empty. A_73_ translocates out of the catalytic pocket, and the position that was occupied by A_73_ at the C_74_-adding stage accommodates the newly incorporated C_74_ (**Figure 6E**). The release of pyrophosphate, a byproduct of RNA polymerization, from the active site of AaS triggers the backward translocation of the tRNA, as observed in T7 RNA polymerase (Yin and Steitz, 2004).

Upon pyrophosphate release from the active site after C_75_-addition, the tRNA translocates further toward the tail domain and rotates relative to the enzyme. A tRNA ending with C_74_C_75_ could no longer be retained on the enzyme, due to the further translocation and rotation of the tRNA relative to the enzyme, and would dissociate after C_74_C_75_ synthesis. Thus, the enzyme would terminate RNA synthesis. The tRNA ending with C_74_C_75_ then binds to AaL, the terminal A_76_ is added, and the CCA-3′ synthesis of tRNA is completed.

Previous biochemical studies using class-II CCA-adding enzymes and a tRNA mini-helix, corresponding to the top-half of tRNA, suggested that C_74_ addition, like C_75_ and A_76_ addition, involves neither tRNA translocation nor rotation (Cho et al., 2006). The mechanisms of CC-addition by the class-II CC-adding andCCA-adding enzymes might be different. Alternatively, the previous biochemical study using a tRNA mini-helix might not represent the actual nature of C_74_-addition onto tRNA, since the tRNA mini-helix lacks the interactions between the TψC loop and the D-loop in tRNA.

**FIGURE 7:**
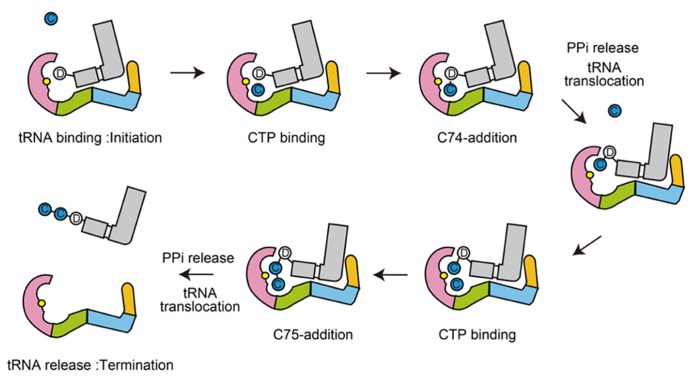
**Mechanism of CC-addition by class-II CC-adding enzymes.** Schematic representation of the CC-adding reaction by class-II CC-adding enzymes. The head, neck, body, and tail domains are colored magenta, green, cyan, and orange, respectively. tRNAs are colored gray. Catalytic sites are colored yellow in the head domains.

The dynamic sequence of the CC-adding reaction by the class-II CC-adding enzyme, revealed by the crystallographic analyses of the complexes of the CC-adding enzyme with various tRNAs with or without and CTP, is presented in **Figure 7**.

## CONCLUSIONS AND PERSPECTIVES

The detailed and extensive crystallographic analyses of the class-I CCA-adding enzyme complexed with various RNA primers explained the previous biochemical results well (Shi et al., 1998a; Yue et al., 1998; Okabe et al., 2003; Xiong et al., 2003; Xiong and Steitz, 2004; Cho et al., 2006; Tomita et al., 2006; Toh et al., 2008; Pan et al., 2010). As previously suggested by biochemical studies using the class-I CCA-adding enzyme (Shi et al., 1998b; Yue et al., 1998; Cho et al., 2006), structural studies of the class-I CCA-adding enzyme revealed that the tRNA neither translocates nor rotates relative to the enzyme during the CCA-adding reaction. The reaction proceeds in a single active pocket, and the template for CTP and ATP is the RNA-protein complex, rather than the protein itself. The crystallographic analyses also showed that the size and the shape of the nucleotide binding pocket, formed by the growing 3′-terminus of the RNA and the enzyme, successively change during the CCA-adding reaction, thus switching the nucleotide specificity, and that the CCA-adding reaction proceeds via two modes – dynamic CC-addition and static A-addition.

On the other hand, regarding the class-II CCA-adding enzyme, only the *apo* structures of the enzyme and its complexes with CTP and ATP are available (Li et al., 2002; Toh et al., 2009). Until now, the structure of a class-II CCA-adding enzyme complexed with tRNA (or RNA) has not been reported, and the detailed molecular mechanism of nucleotide specificity switching during CCA-addition has remained elusive. The complex structures of the class-II CCA-adding enzyme with CTP and ATP revealed that both CTP and ATP are recognized in the same active pocket, through Watson-Crick-like base pairings between the nucleobases and the conserved Asp and Arg residues in the active pocket (Li et al., 2002; Toh et al., 2009). Thus, the template for CTP and ATP is the protein itself, rather than the RNA-protein complex. This is distinct from the mechanism of nucleotide selection by the class-I CCA-adding enzymes.

The complex structures of the *A. aeolicus* class-II**CC-adding and A-adding enzymes with tRNA and a nucleotide were reported (Tomita et al., 2004; Yamashita et al., 2014). The recognition mechanisms of CTP and ATP by the *A. aeolicus* CC-adding and A-adding enzymes at the insertion stage of RNA polymerization, respectively, are the same as those of the class-II CCA-adding enzyme, as revealed by the complex structures of these enzymes with nucleotides (Li et al., 2002; Toh et al., 2009).

The detailed molecular basis for the different activities between the A-adding enzyme and the CC-adding enzymes is not fully understood. The mechanism by which the CC-adding enzyme adds only C_74_C_75_, and then terminates RNA polymerization without adding A_76_, was explained well by the structural analyses (Yamashita et al., 2014). However, the mechanism by which the A-adding enzyme adds only A_76_, but not C_74_C_75_, has not been clarified yet, even though the complex structure of the *A. aeolicus* A-adding enzyme with tRNA and a nucleotide analog is available (Tomita et al., 2004).

The short loop in the head domain is suggested to be a hallmark of the CC-adding enzymes (Neuenfeldt et al., 2008). However, transplantation of the corresponding flexible longer loop from the A-adding enzyme (or CCA-adding enzyme) into the corresponding position of the CC-adding enzyme did not always transform the CC-adding enzyme into a CCA-adding enzyme, *in vitro *as well as* in vivo *(Toh et al., 2009). Thus, the longer loop in the head domain of the A-adding enzyme itself is not the main determinant for the A-adding enzyme to add only terminal A_76_. The C-terminal body and tail domains of the A-adding enzymes reportedly inhibit C_74_C_75_ addition *in vitro* (Tretbar et al., 2011). Since the overall structures of *A. aeolicus* A-adding enzyme and *T. maritima* CCA-adding enzyme superimposed well, the inhibitory effects of the C-terminal region of all A-adding enzymes on CC-addition probably do not dictate the specificity for the enzymes to add only terminal A_76_, in general.

**FIGURE 8:**
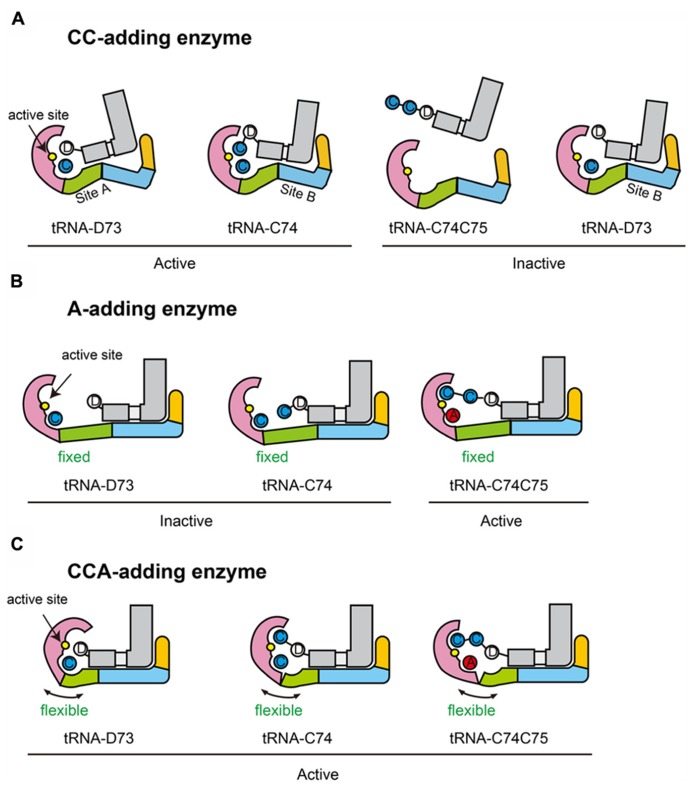
**Hypothetical mechanisms of nucleotide addition by class-II enzymes.** **(A)** CC-addition by CC-adding enzyme possessing two tRNA binding sites (sites A and B). Sites A and B are used for C_74_- and C_75_-addition, respectively. When a tRNA ending with the discriminator nucleoside (D_73_) is on site B, the 3′-terminal D_73_ cannot reach the active site for catalysis (inactive state). A tRNA ending with C_74_C_75_ cannot bind either site A or B. **(B)** A-addition by A-adding enzyme, possessing a single tRNA binding site. The neck domain is not flexible. The tRNA binds the enzyme, using the single site. On this site, the 3′-terminal nucleoside of a tRNA ending in either a discriminator nucleoside (tRNA-D_73_) or C_74_ (tRNA-C_74_) cannot reach the active site (inactive state). Only the 3′-terminal nucleoside of tRNA ending in C_74_C_75_ can reach the active site for the catalysis (active state). **(C)** CCA-addition by CCA-adding enzyme, possessing a single tRNA binding site. tRNA binds the enzyme, using the single site. Since, unlike the A-adding enzyme, the neck domain of the CCA-adding enzyme is flexible, the head domain of the CCA-adding enzyme could relocate toward the neck domain to catalyze C_74_ and C_75_ addition. The head, neck, body, and tail domains of the enzymes are colored magenta, green, cyan, and orange, respectively. tRNAs are colored gray. Catalytic sites are colored yellow in the head domains.

The C_74_C_75_-addition by the CC-adding enzyme involves the translocation and rotation of the tRNA relative to the enzyme (Yamashita et al., 2014). Apparently, there are two tRNA binding sites on the surface of *A. aeolicus *CC-adding enzyme. One is for C_74_-addition and the other is for C_75_-addition (**Figure 8A**). The body domain of *A. aeolicus* CC-adding enzyme adopts a bulging structure and the overall structure has a more closed conformation, as compared with the *A. aeolicus* A-adding enzyme and other CCA-adding enzymes (Li et al., 2002; Tomita et al., 2004; Toh et al., 2009; Yamashita et al., 2014). Thus, the distinct structures of the body domain of the CC-adding enzyme, with two tRNA binding sites, allow the tRNA to translocate and rotate during the CC-adding reactions and to terminate the RNA polymerization after CC-addition.

A chimeric enzyme of *A. aeolicus* A-adding enzyme and the closely related *T. maritima* CCA-adding enzyme (Tomita and Weiner, 2001, 2002), designed based on their crystal structures, was constructed, and the A-adding enzyme was transformed into an enzyme that could perform CCA-addition *in vitro* as well as *in vivo* (Toh et al., 2009). These biochemical and genetic analyses suggested the importance of the flexibility of the neck domain, in defining the number of nucleotides added onto the 3′-end of the tRNA and the nucleotide specificity by the class-II CCA-adding enzyme.

It could be hypothesized that only a single tRNA binding site exists in both the class-II A-adding and CCA-adding enzymes (**Figures 8B,C**). On the single tRNA binding site, the 3′-terminus of tRNA lacking CCA or CA could not reach the active site without structural change of the enzyme, while the 3′-terminus of tRNA lacking A_76_ could reach it. The absence of flexibility in the neck domain of the A-adding enzyme would not allow the head domain of the enzyme to relocate toward the neck domain for CC-addition, when tRNA binds the single tRNA binding site. Hence, the A-adding enzyme could not add C_74_C_75_, but could only add A_76_ (**Figure 8B**). On the other hand, the CCA-adding enzyme, with a flexible neck domain, could add CC as well as A, by relocating the catalytic active head domain toward the neck domain for catalysis (**Figure 8C**), even though there is only one tRNA binding site, as in the A-adding enzyme. In these models, neither translocation nor rotation of tRNA is involved in the CCA-addition by the class-II CCA-adding enzyme, as previously suggested (Cho et al., 2006).

In the future, the complex crystal structures of a class-II CCA-adding enzyme with various tRNAs, representing sequential CCA-addition, and the comparison of the structures with those of the A-adding enzyme and the CC-adding enzyme complexed with tRNAs, will provide clear and definitive answers to all of the above-mentioned unsettled questions about the mechanisms of class-II CCA-adding enzyme and its relatives.

## Conflict of Interest Statement

The authors declare that the research was conducted in the absence of any commercial or financial relationships that could be construed as a potential conflict of interest.

## References

[B1] Aebi M. Kirchner G. Chen J. Y. Vijayraghavan U. Jacobson A. Martin N. C. (1990). Isolation of a temperature-sensitive mutant with an altered tRNA nucleotidyltransferase and cloning of the gene encoding tRNA nucleotidyltransferase in the yeast *Saccharomyces cerevisiae*. *J. Biol. Chem.* 265 16216–16220 2204621

[B2] Augustin M. A. Reichert A. S. Betat H. Huber R. Mörl M. Steegborn C. (2003). Crystal structure of the human CCA-adding enzyme: insights into template-independent polymerization. *J. Mol. Biol.* 328 985–994 10.1016/S0022-2836(03)00381-4 12729736

[B3] Bard J. Zhelkovsky A. M. Helmling S. Earnest T. N. Moore C. L. Bohm A. (2000). Structure of yeast poly(A) polymerase alone and in complex with 3′-dATP. *Science* 289 1346–1349 10.1126/science.289.5483.1346 10958780

[B4] Bralley P. Chang S. A. Jones G. H. (2005). A phylogeny of bacterial RNA nucleotidyltransferases: *Bacillus halodurans* contains two tRNA nucleotidyltransferases. *J. Bacteriol.* 187 5927–5936 10.1128/JB.187.17.5927-5936.2005 16109934 PMC1196141

[B5] Brautigam C. A. Steitz T. A. (1998). Structural and functional insights provided by crystal structures of DNA polymerases and their substrate complexes. *Curr. Opin. Struct. Biol.* 8 54–63 10.1016/S0959-440X(98)80010-9 9519297

[B6] Cho H. D. Chen Y. Varani G. Weiner A. M. (2006). A model for C74 addition by CCA-adding enzymes: C74 addition, like C75 and A76 addition, does not involve tRNA translocation. *J. Biol. Chem.* 281 9801–9811 10.1074/jbc.M512603200 16455665

[B7] Cho H. D. Verlinde C. L. M. J. Weiner A. M. (2007). Reengineering CCA-adding enzymes to function as (U, G)- or dCdCdA-adding enzymes or poly(C, A) and poly(U, G) polymerases. *Proc. Natl. Acad. Sci. U.S.A.* 104 54–59 10.1073/pnas.0606961104 17179213 PMC1765476

[B8] Deutscher M. P. (1982). tRNA nucleotidyltransferase. *Enzymes* 15 183–215 10.1016/S1874-6047(08)60279-6

[B9] Deutscher M. P. (1990). Transfer RNA nucleotidyltransferase. *Methods Enzymol.* 181 434–439 10.1016/0076-6879(90)81141-G 2199760

[B10] Green R. Noller H. F. (1997). Ribosomes and translation. *Annu. Rev. Biochem.* 66 679–716 10.1146/annurev.biochem.66.1.679 9242921

[B11] Holm L. Sander C. (1995). DNA polymerase beta belongs to an ancient nucleotidyltransferase superfamily. *Trends Biochem. Sci.* 20 345–347 10.1016/S0968-0004(00)89071-4 7482698

[B12] Hou Y. M. (2000). Unusual synthesis by the *Escherichia coli* CCA-adding enzyme. *RNA* 6 1031–1043 10.1017/S1355838200000686 10917598 PMC1369978

[B13] Kim D. F. Green R. (1999). Base-pairing between 23S rRNA and tRNA in the ribosomal A site. *Mol. Cell* 4 859–864 10.1016/S1097-2765(00)80395-0 10619032

[B14] Li F. Wang J. Steitz T. A. (2000). *Sulfolobus shibatae* CCA-adding enzyme forms a tetramer upon binding two tRNA molecules: a scrunching-shuttling model of CCA specificity. *J. Mol. Biol.* 304 483–492 10.1006/jmbi.2000.4189 11090289

[B15] Li F. Xiong Y. Wang J. Cho H. D. Tomita K. Weiner A. M. (2002). Crystal structures of the *Bacillus stearothermophilus* CCA-adding enzyme and its complexes with ATP or CTP. *Cell* 111 815–824 10.1016/S0092-8674(02)01115-7 12526808

[B16] Martin G. Keller W. (1996). Mutational analysis of mammalian poly(A) polymerase identifies a region for primer binding and catalytic domain, homologous to the family X polymerases, and to other nucleotidyltransferases. *EMBO J.* 15 2593–2603 8665867 PMC450192

[B17] Martin G. Keller W. (2007). RNA-specific ribonucleotidyl transferases. *RNA* 13 1834–1849 10.1261/rna.652807 17872511 PMC2040100

[B18] Martin G. Keller W. Doublié S. (2000). Crystal structure of mammalian poly(A) polymerase in complex with an analog of ATP. *EMBO J.* 19 4193–4203 10.1093/emboj/19.16.4193 10944102 PMC302044

[B19] Neuenfeldt A. Just A. Betat H Mörl M. (2008). Evolution of tRNA nucleotidyltransferases: a small deletion generated CC-adding enzymes. *Proc. Natl. Acad. Sci. U.S.A.* 105 7953–7958 10.1073/pnas.0801971105 18523015 PMC2430343

[B20] Nissen P. Hansen J. Ban N. Moore P. B. Steitz T. A. (2000). The structural basis of ribosome activity in peptide bond synthesis. *Science* 289 920–930 10.1126/science.289.5481.920 10937990

[B21] Okabe M. Tomita K. Ishitani R. Ishii R. Takeuchi N. Arisaka F. (2003). Divergent evolutions of trinucleotide polymerization revealed by an archaeal CCA-adding enzyme structure. *EMBO J.* 22 5918–5927 10.1093/emboj/cdg563 14592988 PMC275420

[B22] Pace N. R. (1997). A molecular view of microbial diversity and the biosphere. *Science* 276 734–740 10.1126/science.276.5313.734 9115194

[B23] Pan B. Xiong Y. Steitz T. A. (2010). How CCA-adding enzyme selects adenine over cytosine at position 76 of tRNA. *Science* 330 937–940 10.1126/science.1194985 21071662 PMC3087442

[B24] Pelletier H. Sawaya M. R. Kumar A. Wilson S. H. Kraut J. (1994). Crystal structure of rat DNA polymerase beta: evidence for a common polymerase mechanism. *Science* 264 1930–1935 10.1126/science.7516580 7516581

[B25] Sakon J. Liao H. H. Kanikula A. M. Benning M. M. Rayment I. Holden H. M. (1993). Molecular structure of kanamycin nucleotidyltransferase determined to 3.0Å resolution. *Biochemistry* 32 11977–11984 10.1021/bi00096a006 8218273

[B26] Schimmel P. Yang X. L. (2004). Two classes give lessons about CCA. *Nat. Struct. Mol. Biol.* 11 807–808 10.1038/nsmb0904-807 15332079

[B27] Shi P. Y. Maizels N. Weiner A. M. (1998a). CCA addition by tRNA nucleotidyltransferase: polymerization without translocation? *EMBO J.* 17 3169–3206 10.1093/emboj/17.11.3197 PMC1170658 9606201

[B28] Shi P. Y. Weiner A. M. Maizels N. (1998b). A top-half tDNA minihelix is a good substrate for the eubacterial CCA-adding enzyme. *RNA* 4 276–284 9510330 PMC1369617

[B29] Sprinzl M. Cramer F. (1979). The -C-C-A end of tRNA and its role in protein biosynthesis. *Prog. Nucleic Acid Res. Mol. Biol.* 22 1–69 10.1016/S0079-6603(08)60798-9 392600

[B30] Toh Y. Numata T. Watanabe K. Takeshita D. Nureki O. Tomita K. (2008). Molecular basis for maintenance of fidelity during the CCA-adding reaction by a CCA-adding enzyme. *EMBO J.* 27 1944–1952 10.1038/emboj.2008.124 18583961 PMC2486279

[B31] Toh Y. Takeshita D. Nagaike T. Numata T. Tomita K. (2011). Mechanism for the alteration of the substrate specificities of template-independent RNA polymerases. *Structure* 19 232–243 10.1016/j.str.2010.12.006 21300291

[B32] Toh Y. Takeshita D. Numata T. Fukai S. Nureki O. Tomita K. (2009). Mechanism for the definition of elongation and termination by the class II CCA-adding enzyme. *EMBO J.* 28 3353–3365 10.1038/emboj.2009.260 19745807 PMC2776095

[B33] Tomari Y. Suzuki T. Watanabe K. Ueda T. (2000). The role of tightly bound ATP in *Escherichia coli* tRNA nucleotidyltransferase. *Genes Cells* 5 689–698 10.1046/j.1365-2443.2000.00360.x 10971651

[B34] Tomita K. Fukai S. Ishitani R. Ueda T. Takeuchi N. Vassylyev D. G. (2004). Structural basis for template-independent RNA polymerization. *Nature* 430 700–704 10.1038/nature02712 15295603

[B35] Tomita K. Ishitani R. Fukai S. Nureki O. (2006). Complete crystallographic analysis of the dynamics of CCA sequence addition. *Nature* 443 956–960 10.1038/nature05204 17051158

[B36] Tomita K. Weiner A. M. (2001). Collaboration between CC- and A-adding enzymes to build and repair the 3’-terminal CCA of tRNA in *Aquifex aeolicus*. *Science* 294 1334–1336 10.1126/science.1063816 11701927

[B37] Tomita K. Weiner A. M. (2002). Closely related CC- and A-adding enzymes collaborate to construct and repair the 3’-terminal CCA of tRNA in *Synechocystis* sp. and Deinococcus radiodurans. *J. Biol. Chem.* 277 48192–48198 10.1074/jbc.M207527200 12370185

[B38] Tretbar S. Neuenfeldt A. Betat H Mörl M. (2011). An inhibitory C-terminal region dictates the specificity of A-adding enzymes. *Proc. Natl. Acad. Sci. U.S.A.* 108 21040–21045 10.1073/pnas.1116117108 22167803 PMC3248509

[B39] Weiner A. M. (2004). tRNA maturation: RNA polymerization without a nucleic acid template. *Curr. Biol.* 14 883–885 10.1016/j.cub.2004.09.069 15498478

[B40] Wilusz J. E. Whipple J. M. Phizicky E. M. Sharp P. A. (2011). tRNAs marked with CCACCA are targeted for degradation. *Science* 334 817–821 10.1126/science.1213671 22076379 PMC3273417

[B41] Yamashita S. Takeshita D. Tomita K. (2014). Translocation and rotation of tRNA during template-independent RNA polymerization by tRNA nucleotidyltransferase. *Structure* 22 315–325 10.1016/j.str.2013.12.002 24389024

[B42] Yin Y. M. Steitz T. A. (2004). The structural mechanism of translocation and helicase activity in T7 RNA polymerase. *Cell* 116 393–404 10.1016/S0092-8674(04)00120-5 15016374

[B43] Yue D. Maizels N. Weiner A. M. (1996). CCA-adding enzymes and poly(A) polymerases are all members of the same nucleotidyltransferase superfamily: characterization of the CCA-adding enzyme from the archaeal hyperthermophile *Sulfolobus shibatae*. *RNA* 2 895–908 8809016 PMC1369424

[B44] Yue D. Weiner A. M. Maizels N. (1998). The CCA-adding enzyme has a single active site. *J. Biol. Chem.* 273 29693–29700 10.1074/jbc.273.45.29693 9792681

[B45] Xiong Y. Li F. Wang J. Weiner A. M. Steitz T. A. (2003). Crystal structures of an archaeal class I CCA-adding enzyme and its nucleotide complexes. *Mol. Cell.* 12 1165–1172 10.1016/S1097-2765(03)00440-4 14636575

[B46] Xiong Y. Steitz T. A. (2004). Mechanism of transfer RNA maturation by CCA-adding enzyme without using an oligonucleotide template. *Nature* 430 640–645 10.1038/nature02711 15295590

[B47] Xiong Y. Steitz T. A. (2006). A story with a good ending: tRNA 3’-end maturation by CCA-adding enzymes. *Curr. Opin. Struct. Biol*. 16 12–17 10.1016/j.sbi.2005.12.001 16364630

